# Social Networking Sites and Educational Adaptation in Higher Education: A Case Study of Chinese International Students in New Zealand

**DOI:** 10.1100/2012/289356

**Published:** 2012-04-30

**Authors:** Ling Cao, Tingting Zhang

**Affiliations:** ^1^School of Economics and Management, Nanjing University of Information Science and Technology, 210044 Nanjing, China; ^2^Institute of Information and Mathematical Sciences, Massey University, 0745 Auckland, New Zealand

## Abstract

This study aims to find out the relationship between the use of SNSs and educational adaptation process of Chinese international students (from China) in New Zealand. Based on interview data, this paper addressed how Chinese international students use SNSs (RenRen, Facebook, etc.) to expand and manage their online social networks to help their adaptation to new educational environment. As a case study of Chinese international students in New Zealand and from the narrative of students, we examined the relationship among educational difficulties, life satisfaction, and the use of SNSs. This study would help in further understanding how and why SNSs can be adopted in higher education to support effective overseas learning experiences.

## 1. Introduction

International students make valuable educational and economic contributions to New Zealand [1]. The number of Chinese international students has been more than that of other countries in New Zealand for several years since 2001. In 2009, the number of Chinese international students enrolled in New Zealand higher education institutions were 9789 [2]. Despite the high numbers, Chinese international students face many difficulties adapting to New Zealand cultural and academic life. The difficulties they have experienced included language difficulties, differences in education system and a loss of established social networks [3].

Over the past few years, Social networking sites (SNSs hereafter) have attracted a massive following. Given the global growth and the highly social nature of SNSs, the social relationships of Chinese international students might be influenced by SNSs. Chinese international students use SNSs to connect with their social networks both in New Zealand and China. These interactions allow them to become familiar with and alert to their educational system differences and help them to adjust in their new environment. SNSs, especially Facebook, RenRen (formerly well known as Xiaonei, is considered to be the Chinese Facebook), and Skykiwi (New Zealand local Chinese Website) to some extent, enable students to maintain previous connections and build new relationships during their life transitions. SNSs can play an important role in assisting students to expand and manage their online social network [4]. However, little is known about the exact relationship between educational adaptation of Chinese international students and the use of SNSs.

The purpose of the present study is to explore the relationship between the use of SNSs among Chinese international students in New Zealand and their educational adaptation. To explore this issue, Section 2 of this paper presents a literature review of relevant studies.  Section 3 describes the research hypotheses about the use of SNSs as related to Chinese students' educational adaptation. Method is discussed at Section 4, followed by the data analysis and results, implications, and conclusion of this paper.

## 2. Literature Review

This section will address the former research about Chinese international students, SNSs, and adaptation.

### 2.1. Chinese International Students in New Zealand

The New Zealand Tertiary Education Commission has conducted two sectorwide surveys of international students' experiences [5]. There are a number of key findings consistent across both surveys, which will offer a useful framework against which to measure students' feedback from this project, including factors which influenced the decision to study in New Zealand, and ratings of satisfaction regarding aspects of study and social life in this country [6]. The New Zealand Ministry of Education's commissioned study by the University of Waikato revealed a huge gap between Chinese students' expectations and what they really perceived, which is related to social and cultural differences that made it difficult for them to adapt [7].

### 2.2. SNSs and International Students

The extreme popularity and rapid growth of SNSs represent a unique opportunity to enhance the interactions of students to a deeper understanding of different culture. University students spend a significant amount of their time on these websites in order to communicate and socialize. According to a survey of college students in the USA, 68% of the students surveyed use SNSs at least one hour a day [8]. The users log into SNSs from their PCs, laptops, or mobile devices.

Chinese students encounter cultural differences, academic challenges, and psychological and social adjustments [1]. SNSs provide a forum for students and faculty to communicate informally about both educational and personal issues. The formation of SNSs groups for exchanging ideas, concerns, and progress may be beneficial. A mechanism could be provided to promote multicultural interaction by SNSs, such as Facebook. Bowers-Campbell claimed that “not only does Facebook facilitate a connection between students and instructors; it also provides a mechanism for building peer support among students” [9, page 81].

### 2.3. SNSs and Educational Adaptation

Advances in ICT are changing the way people meet and communicate. Online communication technologies can weaken geographical constraints and change personal interactions. Among the many new modes of online communication, SNSs have received growing attention from researchers regarding their effects on people's social capital, psychological well-being, and users' gratifications and social outcomes [10]. SNSs such as Facebook, Myspace, and LinkedIn are examples of widely popular networks used to find and organize contacts. Huijser argued that social network sites could offer great potential for supporting students' learning in higher education [11].

The social relationships between individuals play a crucial role in determining the way problems are solved, and the degree to which individuals succeed in achieving their goals. Chinese international students are tackling a great range of challenges, from academic ones to personal ones. Previous studies showed Chinese students received few social supports from local peers [6]. Ye found that international students received more social support from their interpersonal networks and that those linked to online ethnic social groups reported fewer social difficulties [12]. National School Boards Association (2007) encouraged educators to explore ways to use social networking for educational purposes [13]. Magro et al. argued SNSs could help students learn from each other and enhance social capital in a doctoral education context [4].

In spite of the above research, there is a paucity of research in this area about Chinese international students. Our research provides an initial exploratory study to discuss how SNSs can help Chinese international students to more easily adapt to their educational transition.

## 3. Research Hypotheses

We adopt social network theory as the theoretical lens for our research. From a social network perspective, a social network involves a set of actors and the relations that connect them. Actors (individual people) social network comprises strong ties and weak ties [12]. A tie simply refers to the relationship between a certain individual and a particular network member. Strong ties are more intimate and involve more various forms of resource exchange. The close relationships among strong ties may play an effective role, which can satisfy an individual's needs. For instance, the phenomenon of conational interaction among international students could be observed in class and in common areas such as the canteen and coffee bar [14]. That could be referred to strong ties. Through building and maintaining their conational offline and online support network, they can get access to instrumental support both practically and emotionally. Weak ties, on the other hand, involve fewer intimate exchanges and less frequent maintenance. That means that weak tie relationships allow individuals to be involved in diversifying connections to get alternative social support, such as information exchange, more opportunities for interaction, especially from online communities. According to Ye [12], online communication can foster the development of weak ties. Social network theory is applicable to the examination of social support networks of international students.

Thus, our aim with this study was to explore the educational adaptation of Chinese international students', in particular, how this is related to the use of SNSs. As for SNSs, a fastest-growing and most popular technology with young people is their opportunity to be adopted to accelerate the education adjustment for students?

*RQ1: What kinds of difficulties do Chinese international students encounter when they have life transition?*

Additionally, Students obtained social support through personal interactions of SNSs. This allowed them to reduce uncertainty in a new surrounding and could provide rational feedback and knowledge. Therefore, the opportunity to socialize actively online upon arrival in New Zealand could ease the stress inherent in their transitions.

*RQ2: How Chinese international students achieve greater success with their study abroad experience with aid of SNSs?*

## 4. Method

### 4.1. Method

Semistructured interviews which were conducted in focus group interviews are the main data collection instruments. We examined educational adaptation in Chinese students by asking them questions regarding their experiences in orienting, adjusting, and adapting to the study of life in New Zealand. It is expected that the mode of research, relying on interaction within a group through debates and arguments, will generate new ideas and data across a range of experiences and opinions. The study examined the adjustment issues by drawing on the participants' narrative of their perceptions of the educational adaptation. To obtain reliable and impartial data, interviews were conducted in Mandarin. We believe that if the participants speak in their own language, they would feel at ease in expressing their ideas clearly [15]. This was conducted in a relaxed atmosphere where the participants could express their opinions freely without linguistic constraint. The first author was a visiting Chinese scholar. She did not mark the students' work and had no input in academic assessment. The interviews were audio-taped, transcribed, and translated into English.

The interview questions addressed their learning experience, academic difficulties, and use of SNSs, as follows: what challenges do you face in the new educational environment; do you enjoy or satisfy with your life in New Zealand; how do you expand your support networks on campus and outside; how often do you use SNSs; how do you interact with home and local friends online and offline; do you feel SNSs can help you transmit in your learning experience? and so forth.

### 4.2. Participants

Participants were 25 Chinese international students at a midsize New Zealand university. Participants ranged from 18 to 27 years old with 10 men and 15 women. They were recruited through a direct invitation on the campus.

## 5. Data Analysis

This section will report the finding from the survey.

### 5.1. Chinese International Students Adaptation Difficulties in New Zealand

All the participants have used more than one kind of SNSs and most of them reported having educational adaptation difficulties. Students have particularly intense emotional experiences at the start of academic life when they would be attempting to adapt to the unfamiliar academic situation. Students' support networks play a crucial role to provide mutual assistance from Chinese students. Thus, these would alleviate adjustment problems in the early stage:

*“I have a hard time adjusting at first in New Zealand, although it's nice to come and have chance to be an international undergraduate student in this university.” (Alice)*

As these Chinese international students adjust to new surroundings in New Zealand, all may be confronted with the following similar challenges and problems, such as, language barriers, academic demands, and lack of study skills. They usually have to reestablish their social networks so as to obtain useful suggestions:

*“When I first arrived in New Zealand, everything was new for me. What should I learn about the university? Which courses should I choose? Which paper is proper for my major? All the tips would be received from other Chinese students. Our Chinese students have to depend on each other, because we would understand all the difficulties other students meet and give advises or suggestions.” (Dong)*

### 5.2. Use of SNSs: Seeking Instrumental Support of Education

Using of SNSs can lead to valuable outcomes, such as the building and maintaining of social support networks to ease Chinese international students' pressure from new academic settings. The implicit access to instrumental support allowed Chinese international students to feel supported both practically and emotionally [16]. Access to practical support in everyday life was one of explanation for the use of SNSs, as the following typical comments reveal:

*“Those friends (online) help me a lot, because they show me the way they had experienced, and help me adapt more,… how to do something, how to get through something, and how to have fun in New Zealand. Besides my parents, they support me since I have been here.” (Jiang)*

But not all respondents saw SNSs as a viable source for help in educational adaptation:

*“It was not helpful to me. When I needed academic information, it was easier to look for assistant from university office. There was always someone who could help me. Otherwise, I would like take with my lectures. In spite that there was language barrier, it was still a good way to get direct information about my paper.” (Zheng)*

### 5.3. Use of SNSs: Pooling of Information

SNSs, as an important communication tool, such as QQ, RenRen, through the instant message functions, can provide more chances to encourage and assure Chinese students in a time of life transition. For example, Li said:

*“Using SNSs helped me know more about my university. Facebook groups and events provided me more opportunities to share information with Kiwi students.”*

### 5.4. Use of SNSs: Communication with Friends

The majority of participants utilized the SNSs to maintain close relationships with a small number of ties instead of creating new connections with people:

*“The most important thing about using SNSs is that it helps me keep in touch with my friends. Having large friend numbers online, I feel safe and dependence when I have difficulties.” (Jane)*

*“Why cannot make friends with kiwi students by Facebook? … I do not need many new friends. All my current friends are really helpful. We will discuss our paper through QQ paper group. We will share writing assignment tips when needed. Such things lead to a close relationship group among Chinese international students.” (Lily)*

## 6. Conclusion and Implications

As Ellison et al. pointed out, it is important to study how people use SNSs to manage their social networks when they face life transitions [17]. Based on the interview, the study investigates the relationship between the use of SNSs and Chinese international students' educational adaptation. Our study demonstrated that SNSs can lead to building social networks of Chinese international students to support their study overseas. Results of this small-scale interview in one location indicated students differ somewhat in their current uses of SNSs. Facebook, as the most popular used in western young people, is perceived as the second choice for Chinese international students to build social networks. Several significant findings help to better understand these differences.

The relationships among students in offline contexts have been mirrored online. Regarding SNSs behaviour, most of Chinese international students' online friends were people who already know each other from previous face-to-face interaction [18]. They use the SNSs as a way of keeping their existing social connections alive, rather than for making new friends.

We can imply that SNSs may become yet the technology that had great potential for improving the higher education experience. University as a diverse community should foster diversity social networks to provide mutual assistance, including the pooling of information. That will alleviate adjustment problems in Chinese international students. International campus, as a mixed-nationality setting, should be an ideal place to foster multicultural response. By means of either online or offline cross-cultural interaction, it is time that Higher Education Institutions took some responsibility for encouraging students to utilize the opportunity to grow their international vision.

Findings from this study are potentially useful for education administrators and preparing students for study overseas. It is important for education administrators in universities to realize that they should make an effort to provide more chances for international students' interaction with local students to provide opportunities to develop weak tie relationships. With weak ties established, there will be more opportunities for international students to develop strong ties that can last a life time. Thus, this study should be useful to university administrations and education policy makers when considering Chinese students adapting to western university life.

## 7. Limitation and Future Work

There are several limitations of this study. First, as a qualitative study, the sample size was small which limited the statistical power to generalize. Secondly, it is also quite possible exist positive bias that the respondents in favour of positive attitude of SNSs experiences would like to evolve individual interactions online. Future work in this area should seek to include larger numbers of participants to avoid the potential bias in the results. And the findings through multiple methods will also result in more detailed data.
